# Evolutionary, comparative, and functional analyses of STATs and regulation of the JAK-STAT pathway in lumpfish upon bacterial and poly(I:C) exposure

**DOI:** 10.3389/fcimb.2023.1252744

**Published:** 2023-09-22

**Authors:** Shreesha S. Rao, Patrick A. Nelson, Harald S. Lunde, Gyri T. Haugland

**Affiliations:** Department of Biological Sciences, Bergen High-Technology Centre, University of Bergen, Bergen, Norway

**Keywords:** lumpfish, JAK-STAT, *Vibrio anguillarum*, Poly(I:C), lumpsucker, transcriptome

## Abstract

**Background:**

The Janus kinase/signal transducers and activators of transcription (JAK-STAT) system regulates several biological processes by affecting transcription of genes as a response to cytokines and growth factors. In the present study, we have characterized the STAT genes in lumpfish (*Cyclopterus lumpus* L.), belonging to the order Perciformes, and investigated regulation of the JAK-STAT signaling pathway upon exposure to bacteria (*Vibrio anguillarum*) and poly(I:C), the latter mimicking antiviral responses.

**Methods:**

Characterization and evolutionary analyses of the STATs were performed by phylogeny, protein domain, homology similarity and synteny analyses. Antibacterial and antiviral responses were investigated by performing KEGG pathway analysis.

**Results:**

We observed that lumpfish have *stat1a*, *2*, *3*, *4*, *5a*, *5b*, and *6*. Transcriptome-wide analyses showed that most components of the JAK-STAT pathway were present in lumpfish. *il*-6, *il*-10, *il*-21, *iκBα* and *stat3* were upregulated 6 hours post exposure (hpe) against bacteria while type I interferons (IFNs), *irf1*, *irf3*, *irf10*, *stat1* and *2* were upregulated 24 hpe against poly(I:C).

**Conclusions:**

Our findings shed light on the diversity and evolution of the STATs and the data show that the STAT genes are highly conserved among fish, including lumpfish. The transcriptome-wide analyses lay the groundwork for future research into the functional significance of these genes in regulating critical biological processes and make an important basis for development of prophylactic measure such as vaccination, which is highly needed for lumpfish since it is vulnerable for both bacterial and viral diseases.

## 1 Introduction

Cytokines promote communication and are important for a range of biological processes, including formation and regulation of immune cells and hematopoietic cells (Zou and Secombes, 2016). The cytokines bind to specific receptors on target cells, triggering a chain reaction of intracellular signaling processes controlled by the Janus kinase/signal transducers and activators of transcription (JAK-STAT) pathway (Morris et al., 2018). JAKs are a family of intracellular non-receptor tyrosine kinases, consisting of four members, *jak1*, *jak2*, *jak3* and *tyk2* (Bousoik and Montazeri Aliabadi, 2018). Activation of the JAKs causes phosphorylation and activation of STATs, which then translocate to the nucleus and bind to DNA regulatory sites to control gene expression (Seif et al., 2017). The JAK-STAT system is a flexible signaling pathway that permits different signaling molecules, such as cytokines, colony-stimulating factors, and hormones, to control gene expression and have impact on a variety of cellular functions (O’Shea et al., 2015). While this pathway was initially identified during research on how interferons (IFNs) regulate gene expression (Levy et al., 1988; Schindler et al., 1992), it is now known also to be important for proper functioning of multicellular organisms, involved in various processes such as maintaining homeostasis, host defense, and cell growth both in humans and fish (Ward and Thompson, 2012; Jin et al., 2018).

Each STAT gene has an N-terminal domain (NTD), which takes role in dimerization, nuclear translocation, and protein interaction. They also have a coiled-coil domain, which aids in protein-protein interactions and receptor binding, a DNA binding domain (DBD), a linker region, an SH2 domain needed for receptor binding and dimerization, and a C-terminal transactivation domain (TAD) that regulates STAT transcriptional activity (Langenfeld et al., 2015). A conserved tyrosine residue is shared by all members of the family and is phosphorylated prior to dimerization (Hu et al., 2021), and most STATs have a second phosphorylation site, conserved serine residue, within a P(M)SP motif in the TAD domain (Decker and Kovarik, 2000). The STAT domains emerged at different stages of evolution, with the coiled-coil domain being observed more prominently in the phylum Chordata (Surkont and Pereira-Leal, 2015).

While different cytokines activate specific STATs, the degree of interactive promiscuity between cytokines and any given STAT varies with vertebrates (Skjesol et al., 2010), including fish. Scientists have made substantial progress in characterizing members of the STAT family, which include STAT1-6 (Xu et al., 2022). Fish also possess two homologues of the STAT1 gene, named *stat1a* and *stat1b*. The origin of this duplication event can be traced back approximately 35 million years, arising from a genome duplication event in fish. Notably, certain fish species, including salmonids, display an even greater number of *stat1* gene copies, ranging from 2 to 5 copies (Skjesol et al., 2010). This highlights the diverse genetic landscape and complexity within the STAT genes across fish species (Volff, 2005; Skjesol et al., 2010; Song et al., 2011; Xu et al., 2022). Similarly, the STAT5 gene is comprised of two homologues, STAT5A and STAT5B, exhibiting 90% similarity at the amino acid level (Maurer et al., 2019). In mammals, these homologues are located adjacently on the same chromosome (Moll et al., 2019), while in fish, *stat5a* and *stat5b* are situated on separate chromosomes, despite high sequence similarity (Gorissen et al., 2011).

Cytokine binding to its cognate receptor induces conformational changes that activate the associated JAKs (Bousoik and Montazeri Aliabadi, 2018). Activated JAKs undergo autophosphorylation and/or transphosphorylation, and subsequently phosphorylating the cytokine receptors (Beirer and Hofer, 2006). The resulting phosphorylated receptor tyrosine motifs act as docking sites for the SH2 domains of STATs (Skjesol et al., 2014). Upon binding, the STATs become tyrosine phosphorylated, dissociate from the receptor, dimerize, translocate to the nucleus, bind to specific DNA elements in gene promoters, and activate transcription (Schindler and Darnell, 1995). The activation of the JAK-STAT pathway in zebrafish occurs when IFNs bind to their respective receptors (IFNRs), resulting in the transcription of IFN-stimulated genes (ISGs) (Langevin et al., 2013b).

The induction of *stat1* and *stat2* genes has been observed in diverse fish species in response to viral infections, suggesting their participation in antiviral defense mechanisms, mirroring their mammalian counterparts (Gorissen et al., 2011; Dehler et al., 2019). In all cases the genes linked to the JAK-STAT pathway, including *stat1*, *stat2*, IFNs, and IFN regulatory factors (IRFs), show increased expression after poly(I:C) treatment. Other STATs participate in antibacterial responses. Investigations in common carp and Nile tilapia have revealed that the interleukin IL-22 is important in regulating inflammatory reactions by way of the JAK-STAT signaling pathway through STAT3 (Wang JX. et al., 2022; Wang X. et al., 2022). STAT3 can also be activated by IL-6 (Wang JX. et al., 2022). IL-10 is an anti-inflammatory cytokine, mediating the response through STAT3 (Cevey et al., 2019). IL-4 and IL-13 are regulators of T helper 2 (Th2) cell development signaling through STAT6 (Ranasinghe et al., 2018). Teleosts have cytokines related to both and are termed IL-4/IL-13 (Wang and Secombes, 2015; Stocchi et al., 2017). Although the subsets of STATs are conserved within vertebrates (Liongue et al., 2012), it is important to note that fish, particularly salmonids, have several paralogues of the different STATs (Sobhkhez et al., 2014), likely due to the third round of whole genome duplication (Glasauer and Neuhauss, 2014).

Model systems like zebrafish will continue to offer valuable insights into the JAK-STAT pathway and other immunological signaling pathways. However, to fully understand immunological responses in lumpfish, it is crucial to unveil their genetic distinctions in comparison to other fish species (Eggestøl et al., 2020a; Eggestøl et al., 2020b). In our previous studies in lumpfish, early immune responses upon exposure to bacteria and poly(I:C) have been described (Eggestøl et al., 2018; Rao et al., 2023). Upon bacterial encounter, the soluble version of toll like receptor 5 (TLR5s) and proinflammatory cytokines were among the most highly upregulated genes (Eggestøl et al., 2018), while upon poly(I:C) stimulation, the RLR family members MDA5 and LGP2, as well as type I IFNs were highly upregulated (Rao et al., 2023). However, characterization of STATs has not been addressed so far. This research article presents a comprehensive analysis of previously published data to identify and characterize STATs and the JAK-STAT signaling pathway in lumpfish (Eggestøl et al., 2018; Rao et al., 2023), unveil their immunological importance, as well as shed light on the evolution of STATs.

## 2 Materials and methods

### 2.1 Fish and rearing conditions

Unvaccinated, farmed lumpfish were provided by Fjord Forsk Sogn AS (a commercial breeder in Sogn & Fjordane County, Norway), and kept in a 500 L tank in the rearing facilities at the Industrial and Aquatic Laboratory (ILAB) at Bergen High Technology Centre under normal rearing conditions, with optimum temperature oxygen level and salinity. The fish were fed with dry commercial feed. In accordance with Norwegian law, the fish were sacrificed by receiving a sharp blow to the head. All experiments were conducted in accordance with the necessary regulations and guidelines. Under Norwegian law, raising fish in normal, ideal conditions is not subject to ethical review (FOR 1996- 01- 15 no. 23).

### 2.2 Isolation of leukocytes and *in vitro* stimulation

The head kidney was dissected aseptically from lumpfish and leukocytes were isolated using discontinuous Percoll gradient as described earlier (Haugland et al., 2012), (n=15 fish). The quality and quantity of leukocytes were determined using a CASY cell counter, and 5 x 10^6^ leukocytes were added per well in 24-well plates. For bacteria exposure, leukocytes were exposed to *V. anguillarum* serotype O1, multiplicity of infection (MOI) of 1:10, for 6 and 24 hours (hrs) as described in Eggestøl et al (Eggestøl et al., 2018). To monitor anti-viral responses, 100 µg mL-1 poly(I:C) were added to 5 x 10^6^ leukocytes in 24-well plates and incubated for 6 and 24 hrs as described in Rao et al. (2023). For negative controls, leukocytes with L-15+medium were used instead of bacteria (*V. anguillarum*) or poly(I:C).

### 2.3 RNA isolation and RNA sequencing

RNA was isolated using GeneElute Mammalian Total RNA miniprep kit (Sigma). The quality and quantity were determined by bioanalyzer. Prior to RNA sequencing, an equal amount (1 µg) RNA from leukocytes isolated from five fish were pooled (Eggestøl et al., 2018; Rao et al., 2023), and in total there were three parallels in the RNA sequencing.

### 2.4 Differential expression analysis

Normalized read counts analyses which make the basis for the differential expression analysis are described in Eggestøl et al. (2018) and Rao et al. (2023), and the read count files has been submitted to Array express, accession numbers E-MTAB-6388 (*V. anguillarum*) and E-MTAB-12884 (poly(I:C)), respectively.The set of differentially expressed genes (DEGs) comprised those genes that demonstrated statistical significance, with a p-value below 0.05, as determined by our analysis (Eggestøl et al., 2018; Rao et al., 2023).

### 2.5 Multiple sequence alignment and phylogenetic analyses

The lumpfish transcriptomes from leukocytes exposed to *V. anguillarum* (E-MTAB-6388 (Eggestøl et al., 2018)) and poly(I:C) (E-MTAB-12884 (Rao et al., 2023)) was utilized to isolate the sequences of the STAT candidate genes, encompassing the open reading frame (ORF) as well as the untranslated regions. The sequence alignment of the STATs was performed in MEGA 11 (Tamura et al., 2021) using the MUSCLE program with default parameters. The best-fitting models of sequence evolution were determined with complete deletion of gaps. The maximum likelihood method was used to construct the phylogenetic tree using IQ-TREE with automatic model selection and 100,000 bootstraps (Nguyen et al., 2015).

### 2.6 Signaling pathway analyses

Transcriptome-wide differential gene expression (DEG) analyses of the JAK-STAT signaling pathway, Kyoto Encyclopedia of Genes and Genomes (KEGG) map 04630, in head kidney leukocytes following exposure to *V. anguillarum* and poly I:C for 6 and 24 hours (hrs) were conducted using KEGG enrichment analyses (Kanehisa and Goto, 2000; Kanehisa et al., 2012; Kanehisa et al., 2017), and our transcriptomic data submitted to Array Express E-MTAB-6388 (Eggestøl et al., 2018) and E-MTAB-12884 (Rao et al., 2023).

### 2.7 Synteny analysis

Synteny analysis was performed to compare the genomic arrangements of lumpfish STAT genes with those of human (*Homo sapiens*), Atlantic salmon (*Salmo salar*), zebrafish (*Danio rerio*), and stickleback (*Gasterosteus aculeatus*) to identify orthologous genes across species. To visualize the genomic arrangement of the STAT genes in other model organisms, the lumpfish sequences were used as queries in the ENSEMBL (https://www.ensembl.org) database. The chromosomal localization, transcriptional orientation, and neighborhood genes of the orthologous STAT genes in all species were obtained using the genome data viewer of NCBI (https://www.ncbi.nlm.nih.gov) and comparative genomics alignment viewer of genomicus v.109 (https://www.genomicus.bio.ens.psl.eu/genomicus-109.01).

## 3 Results

Comprehensive bioinformatic analyses were performed to identify and characterize the STAT family members in lumpfish, to gain insight into the evolution of the STAT family and regulation of the JAK-STAT signaling pathway upon exposure to bacteria and poly(I:C).

### 3.1 Identification and phylogenetic analyses of STATs in lumpfish.

**Figure 1:**
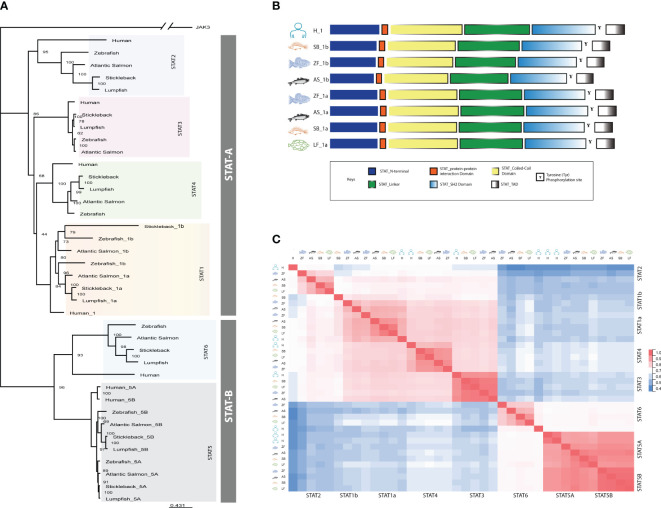
Domain prediction of lumpfish STAT sequences. **(A)** Schematical overview of the different domains. **(B)** Sequence alignment of lumpfish STAT gene family members. Domains are marked with boxes. The domains are defined by *stat1a*. N-terminal domain (NTD, IPR036535), coiled-coil domain (CCD, IPR015988), DNA-binding domain (DBD, IPR013801), src-homology 2 domain (SH-2, IPR000980) and C-terminal transactivation domain (TAD, IPR038295). Conserved regions within the domains are colored. All STAT genes, including *stat1a* (ENSCLMG00005003533), *stat2* (ENSCLMG00005010239), *stat3* (ENSCLMG00005015156), *stat4* (ENSCLMG00005009987), *stat5a* (ENSCLMG00005015196), and *stat6* (ENSCLMG00005021957), were identified and annotated against various scaffolds from the available genome database of lumpfish. **(C)** Estimates of evolutionary divergence between sequences of STAT genes from human (H), Atlantic salmon (AS), zebrafish (ZF), stickleback (SB), and lumpfish (LF) based on a distance matrix.

Seven STAT genes were identified in our transcriptomic data (E-MTAB-6388 and E-MTAB-12884) and STATs candidates in lumpfish were also searched for in Genomicus, Ensembl and NCBI Genbank databases. A phylogenetic tree was constructed to determine the subgroups of lumpfish STATs and the phylogenetic relationship among the groups. Two main clades were made, one with *stat1-4* sequences (STAT-A) and the other clade with *stat5* and *6* (STAT-B). The phylogenetic analyses unveiled that lumpfish have *stat1a*, *2*, *3*, *4*, *5a, 5b* and *6* (**Figure 1A**), and that the lumpfish and stickleback STATs had the highest level of similarity. *stat1b* was not identified in lumpfish. The *stat1b* of stickleback grouped together with *stat1b* sequences from zebrafish and salmon but was distantly related.

All the STATs identified in lumpfish had conserved domains which include N-terminal domain, coiled-coil domain, DNA-linker domain, a linker region, SH2 domain and a C-terminal transactivation domain (**Figure 1B**). The conserved amino acids known to be involved in phosphorylation, S, S and Y, in the C-terminal were present in all the lumpfish sequences (**Supplementary Figure 1**). A multiple sequence alignment of the different STATs showed high similarity within the different domains (**Supplementary Figure 1**). A distance matrix analysis showed the same grouping into STAT-A and STAT-B as the phylogenetic tree (**Figure 1C**). The different STAT members in lumpfish contained conserved domains and phosphorylation sites shared by other STATs from different phyla, highlighting the proteins’ vital role in signal transduction. However, the lack of stat1b in lumpfish unveils species specific adaptation to prevent infections.

### 3.2 Synteny analyses show that the STAT loci are conserved within teleosts.

**Figure 2:**
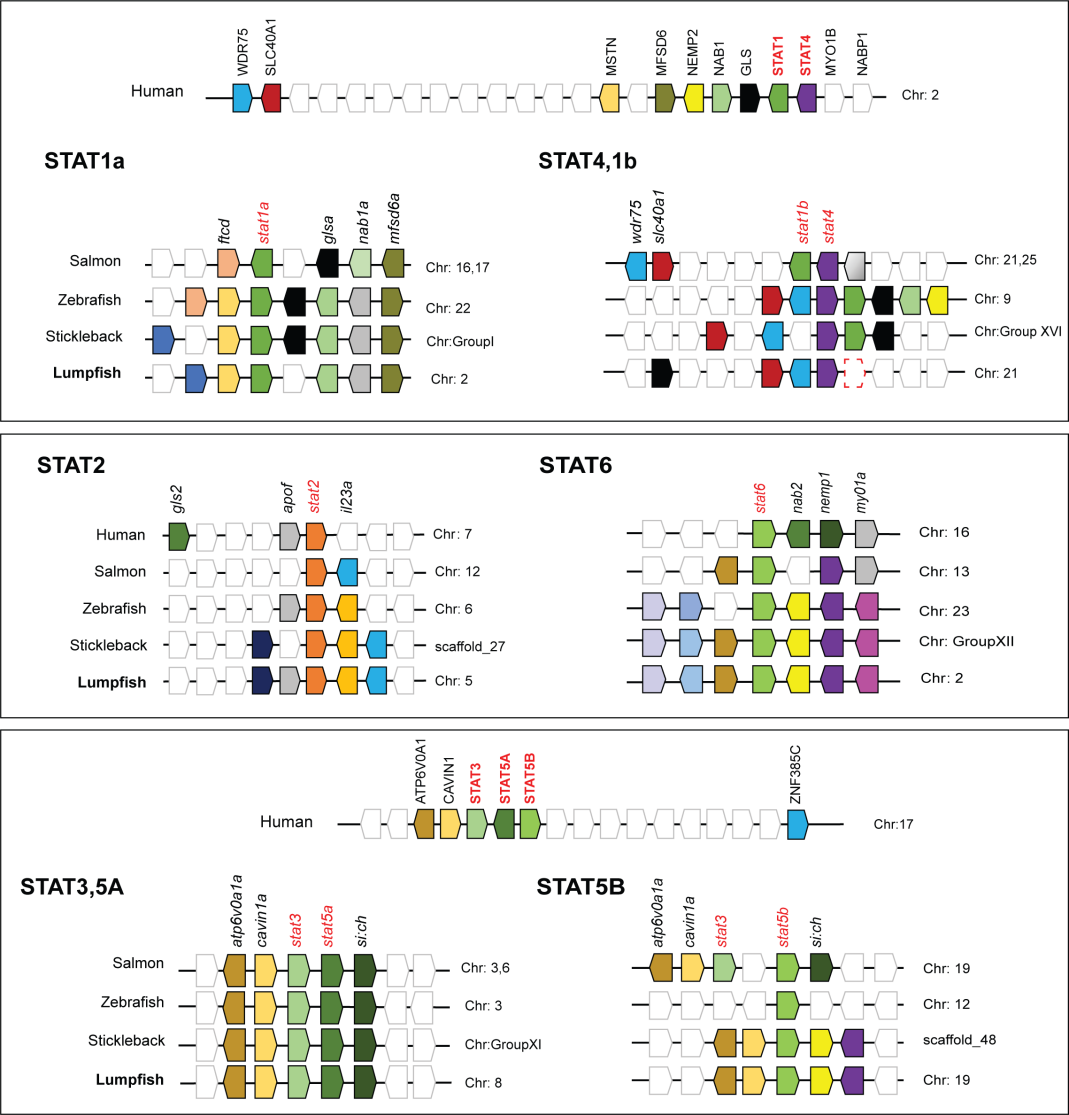
Synteny analyses of human, Atlantic salmon, zebrafish, stickleback, and lumpfish STAT genes. Genomic loci maps comparing STAT related genes were constructed using the Genomicus Browser and NCBI database. The bar lengths are not proportional to the distances between genes. The white boxes represent non-conserved genes on the chromosomes/scaffolds. The direction of the arrows indicates the gene orientation.

Synteny analyses were performed for the STATs (**Figure 2**). In humans STAT1 and STAT4 are located next to each other on chromosome 2. In fish, most species have two copies of *stat1* (a and b), except lumpfish which lack *stat1b*, and it is the *stat1b* that is located next to *stat4* in proximity to *wdr75* and *slc40a1* (**Figure 2**). The lumpfish *stat1a* was found on chromosome 2 in proximity to *mstn* and *nab1a*, like in the other fish species. Both STAT2 and STAT6 had conserved synteny across human and fish (**Figure 2**). In humans, STAT3, STAT5A, and STAT5B are adjacent genes, whereas in fish, they are divided into separate genomic regions. Interestingly, in salmon, the conserved *stat3* gene is duplicated on both chromosomes. However, in zebrafish, stickleback, and lumpfish, the chromosome containing *stat5b* lacks the *stat3* gene, with only a single copy of *stat3* gene present (**Figure 2**). Synteny analyses revealed that the STAT loci were conserved, but in some cases divergent from humans (STAT1 and STAT5A/B), which is not unexpected as fish have gone through an extra round of WGD.

### 3.3 Description of the JAK-STAT pathway and regulation upon *V. anguillarum* and poly(I:C) exposure.

**Figure 3:**
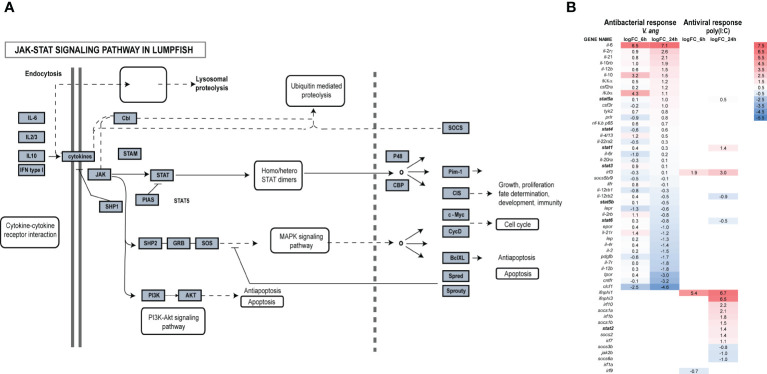
Transcriptome-wide analyses of the JAK-STAT pathway in lumpfish. **(A)** Identified members of the JAK-STAT pathway are shown in grey boxes. The figure has been modified from hsa04630. **(B)** DEG analyses of the JAK-STAT pathway upon exposure to bacteria and poly(I:C) after 6 and 24hpe.

**Figure 4:**
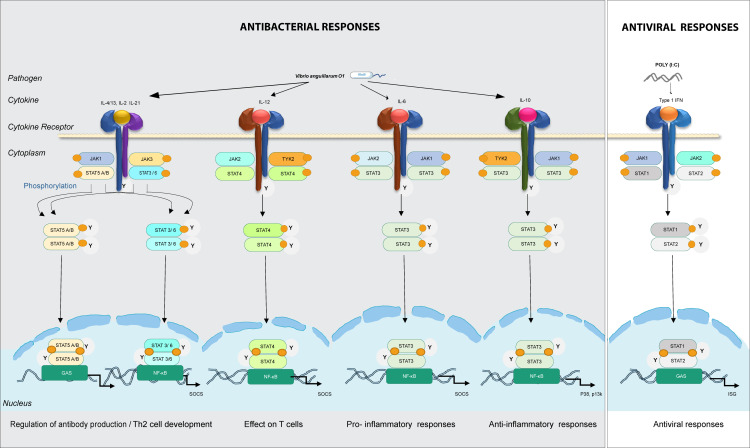
Predicted model for involvement of the different JAKs and STATs family members in intracellular signaling in lumpfish upon bacterial and viral (mimicked by poly(I:C)) encounter. In bacterial responses, *il*-4/13*, il-*21*, il-*6, and *il-*10 bind to their cognate receptors, recruit JAK family members (*jak1*/*2*/*3*/*tyk2*) to interact with the cytokine receptor and thereafter STAT (*stat3*/*6* in bacteria responses). In viral responses, IFNs bind to receptors, recruit *jak1*/*2* and *stat1*/*2*. Predicted immune processes initiated are listed.

Transcriptome and genome data mining for genes belonging to the JAK-STAT pathway, revealed that most genes were present including *jak1*-*3* and *tyk2*, all STATs except *stat1b* (**Figure 1**), suppressor of cytokine signaling (SOCS)1a/b, 3b, 5b/9, 6a and other components of the pathway (**Figure 3A**; **Table 1**). DEG analyses of lumpfish leukocytes exposed to *V. anguillarum* and poly(I:C) were performed in two separate experiments. Notably, exposure to bacteria resulted in high upregulation of *il*-6, and moderate upregulation of *il-*10*, il-*4/13, and *il-*21 (**Figure 3B**). *stat1a* and *3* were weakly upregulated at both 6 and 24 hpe to bacteria, and opposite to *stat4, stat6* was slightly upregulated at 6 hpe, but not at 24 hpe. Upon exposure to poly(I:C), the highest upregulations were seen for *ifnphi1* (5.4 and 6.7 log2fold upregulated at 6 and 24 hpe, respectively) and *ifnphi3* (6.5 log2fold upregulation). *stat1* and *2* were both upregulated at 24 hpe, as also *irf3* (**Figure 3B**). Based on the DEG analyses, we have suggested a model of how JAK and STAT family members are involved in antibacterial and antiviral responses in lumpfish (**Figure 4**)

**Table 1:** Verified genes belonging to the JAK/STAT pathway (KEGG map04630) in lumpfish.

Trinity no (E-MTAB-6388)	KEGG ID	Abbreviation	Name	Ensemble Number	Accession number of top BLAST hits
TR24161+TR80484	K11225	STAT6	Signal transducer and activator of transcription 6	ENSCLMG00005021957	XP_034392524.1
TR28109	K11217	JAK1	Janus kinase 1	ENSCLMG00005015131	XP_034386002.1
TR7188	K04698	SOCS5b	Suppressor of cytokine signaling 5b	ENSCLMG00005022150	XP_034408562.1
TR84931	K04702	PIM-1	Proto-oncogene serine/threonine-protein kinase	ENSCLMG00005009341	XP_034393193.1
TR80028	K04447	JAK2b	Janus kinase 2b	ENSCLMG00005000682	XP_034397336.1
TR18958	K04692	STAT3	Signal transducer and activator of transcription 3	ENSCLMG00005015156	XP_034394600.1
TR81762+ TR65901	K11220	STAT1a	Signal transducer and activator of transcription 1a	ENSCLMG00005003533	XP_034404918.1
TR68888	K16065	PIAS4a	Protein inhibitor of activated STAT, 4a	ENSCLMG00005016833	XP_034386991.1
TR36556	K04705	STAM	Signal transducing adaptor molecule (SH3 domain and ITAM motif) 1	ENSCLMG00005020084	XP_034411665.1
TR26665	K11222	STAT4	Signal transducer and activator of transcription 4	ENSCLMG00005009987	XP_034417089.1
TR64928	K04694	SOCS1a	Suppressor of cytokine signaling 1a	ENSCLMG00005005791	XP_034396207.1
TR49925	K04694	SOCS1b	Suppressor of cytokine signaling 1b	ENSCLMG00005002557	XP_034400876.1
TR67472	K04696	SOCS3b	Suppressor of cytokine signaling 3	ENSCLMG00005021654	XP_034414496.1
TR28557	K04696	SOCS3a	Suppressor of cytokine signaling 3a	ENSCLMG00005015318	XP_034395107.1
TR27289+TR28097+ TR41905+TR8000	K11223	STAT5A	Signal transducer and activator of transcription 5A	ENSCLMG00005015196	XP_034394597.1
TR80476	K11224	STAT5B	Signal transducer and activator of transcription 5B	ENSCLMG00005017037	XP_034414101.1
TR23643	K04706	PIAS2	Protein inhibitor of activated STAT, 2	ENSCLMG00005004977	XP_034398083.1
TR26670	K04706	PIAS1a	Protein inhibitor of activated STAT, 1a	ENSCLMG00005020781	XP_034385276.1
TR26670	K04706	PIAS1b	Protein inhibitor of activated STAT, 1b	ENSCLMG00005019623	XP_034391186.1
TR83643	K04707	CBL	Cbl proto-oncogene	ENSCLMG00005002297	XP_034406720.1
TR52754	K03099	SOS2	Son of sevenless homolog 2	ENSCLMG00005018431	XP_034381239.1
TR81271	K04695	SOCS2	Suppressor of cytokine signaling 2	ENSCLMG00005018701	XP_034381891.1
TR14185	K05443	IL10	Interleukin 10	ENSCLMG00005009817	XP_034388772.1
TR56134	K04456	AKT2	v-akt murine thymoma viral oncogene homolog 2	ENSCLMG00005014826	XP_034403789.1
TR84455	K04456	AKT3b	v-akt murine thymoma viral oncogene homolog 3b	ENSCLMG00005015395	XP_031732488.1
TR144791	K04456	AKT3a	v-akt murine thymoma viral oncogene homolog 3a	ENSCLMG00005004865	XP_034407303.1
TR29239	K04456	AKT1	v-akt murine thymoma viral oncogene homolog 1	ENSCLMG00005015042	XP_034419134.1
TR65314	K04699	SOCS6a	Suppressor of cytokine signaling 6a	ENSCLMG00005014219	XP_034415872.1
TR80028	K11218	JAK3	Janus kinase 3 (a protein tyrosine kinase, leukocyte)	ENSCLMG00005009723	XP_034386481.1
TR70740	K11221	STAT2	Signal transducer and activator of transcription 2	ENSCLMG00005010239	XP_034387690.1
TR87542	K05435/ K05430	IL4/13	Interleukin 4/13	ENSCLMG00005011822	TNN40301.1
TR87818	K05405	IL6	Interleukin 6	ENSCLMG00005022205	XP_034401434.1
TR11160	K05429	IL2	Interleukin 2	ENSCLMG00005012727	NP_001254612.1
TR26681	K04699	SOCS7	Suppressor of cytokine signaling 7	ENSCLMG00005002228	XP_034391487.1
TR7188	K04698	SOCS9	Suppressor of cytokine signaling 9	ENSCLMG00005006238	XP_034404935.1
TR26681	K03099	SOS1	Son of sevenless homolog 1	ENSCLMG00005011965	XP_034385749.1
TR64766	K04447	JAK2a	Janus kinase 2a	ENSCLMG00005009023	XP_034402224.1
TR11160	K04687	IFN-γa	interferon gamma 1-like	ENSCLMG00005019471	XP_054480966.1
TR131687	K04687	IFN-γb	interferon gamma 1-like	ENSCLMG00005019472	XP_034382228.1
TR8399	K05444	IL19	Interleukin 19	ENSCLMG00005009818	XP_034389491.1
TR8399	K05445	IL22	Interleukin 22	ENSCLMG00005019470	XP_034382121.1
TR77194	K05434	IL21	Interleukin 21	ENSCLMG00005012729	XP_034399994.1

IL-2 might have a dual role promoting IL-4 receptor expression directly, and/or it might also activate STAT5A and STAT5B, which then bind to the IL-4 promoter, resulting in its overexpression. Over expression of STAT3 with JAK1, JAK3 and STAT6 can have a pivotal role in IL-4 signal transmission. Similarly, STAT3 may interact extensively with STAT5A/B and STAT6 in the presence of JAK1 and JAK3. It is also evident that STAT signaling is likely critical for pro-inflammatory and anti-inflammatory processes. For instance, both IL-6 and IL-10 engage in activating STAT3. Differentiation of Th1 cells, which is fueled by the overexpression of STAT4 and vitally dependent on TYK2 and JAK2, are triggered by IL-12 signaling. In our model, Type I IFNs lead to activation of STAT1 and STAT2, which are essential for Th1 polarization in antiviral responses. Transcriptome-wide analyses and mapping of signaling pathways is critical to understand the importance of JAKs and STATs in antibacterial and antiviral responses.

### 3.4 Establishment of evolutionary model of STAT genes

**Figure 5:**
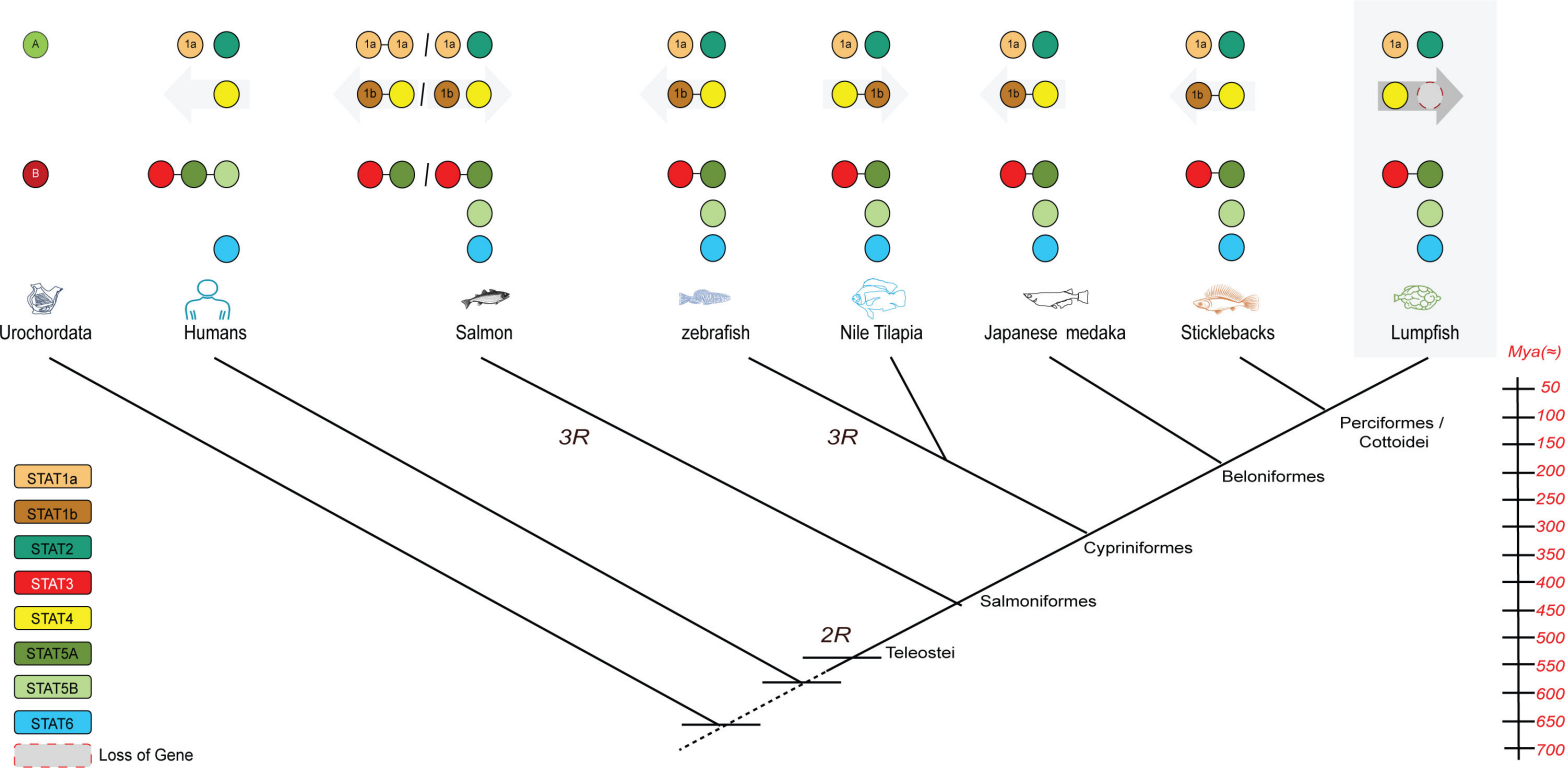
A proposed mechanism for STAT evolution based on 2R hypothesis as a basis of the radiation of the STAT repertoire, the fish-specific whole genome duplication (3R), and subsequent (partial) loss of paralogous *stat1b* genes. Animal diagrams from left to right: human, Atlantic salmon, zebrafish, Nile tilapia, Japanese medaka, stickleback and lumpfish.

Our comparative genomics analysis reveals that STAT genes in fish have undergone multiple duplications throughout evolutionary history (**Figure 5**). The origin of the STAT genes, represented by STAT-A and STAT-B, can be traced back to early chordate to vertebrate evolution. This likely resulted from two whole genome duplication (1R) events after the divergence of cephalochordates (Glasauer and Neuhauss, 2014), but before the emergence of Chondrichthyes. Two rounds (2R) of whole genome duplication during early vertebrate evolution led to the emergence of multiple STAT genes with distinct functions (Boudinot et al., 2021). For instance, the precursor gene STAT-A underwent independent chromosomal duplications, giving rise to STAT1 with STAT4 in one chromosome and STAT2 in another. Similarly, STAT-B underwent independent chromosomal duplications, leading to the formation of STAT3, two copies of STAT5 in one chromosome, and STAT6 in another. In Salmon and Zebra fish species, a third round of whole genome duplication occurred, resulting in the presence of multiple pseudo-genes. Investigating the lumpfish genome, we observed unique gene distribution compared to other fish species. Notably, the lumpfish genome contained *stat1a*, *stat2*, *stat6*, *stat5b*, and *stat4* genes in separate chromosomes, while *stat3* along with *stat5a* was found on a single chromosome (**Figure 5**). Some variations are seen in fish species, e.g., two copies of *stat1a* in salmon and lack of *stat1b* in lumpfish. Several gene duplications in fish STAT genes, dating back to early vertebrate evolution, become apparent in our comparative genomic analyses, highlighting the consequences of WGD events.

## 4 Discussion

The JAK-STAT pathway is a vital signal transduction cascade responsible for regulating diverse biological processes such as immunity, development, and oncogenesis (Seif et al., 2017). The JAK-STAT pathway in fish, particularly the lumpfish system, has been the subject of interest for this study. Lumpfish are widely distributed from the Bay of Biscay in France to the frigid waters of the North Atlantic and Arctic oceans (Whittaker et al., 2018). In recent years, it has become a valuable aquaculture species in Europe and Canada, as they are used as cleaner fish to control sea lice on farmed salmon (Powell et al., 2018; Haugland et al., 2020). The identification of STATs and the JAK-STAT signaling pathway in lumpfish is a major step towards understanding the species’ immunological responses and is important for developing effective disease management strategies for this species. Our findings have provided insights into the activation of this pathway during the immune response to bacterial and viral infections. Due to the complexity and interaction of cytokine signaling pathways, the increasing understanding of the roles of the STATs is important for the development of effective disease therapeutics.

The evolution process describes how organism populations evolve throughout time (Liongue et al., 2012; Liongue et al., 2016). Changes in the heritable traits of biological populations result in genetic variations (Sunyer and Lambris, 1998). Because of recurrent stress followed by reproduction, generations of a single organism can diverge from an ancestral population. Population changes can be so extreme that a new species with a varied range of gene allies is assumed to have developed on rare occasions. Understanding the degree of heterozygosity in neutral evolution is critical for explaining how variations in gene expression over time emerge from divergence accumulation in the absence of selective pressure (Roberts et al., 1998). A crucial stage in the development of the JAK-STAT pathway was the assembly of these STAT domains to produce each individual component. sequence divergence can alter these domains. Instead of creating a domain from scratch the analysis of the individual STAT supported these processes as the primary mechanism for protein domain variation in eukaryotes.

The generation of novel genes in eukaryotes primarily occurs through gene duplications, domain shuffling, and related mechanisms, rather than the creation of entirely new genes (Boudinot et al., 2021). Prior to the first whole genome duplications (WGD), the first generation of the STAT genes underwent tandem duplication (Liongue et al., 2012), giving rise to STAT1/2/3/4 and STAT5/6 (Liongue and Ward, 2013). During the second phase of WGD, three gene clusters were created: STAT1 and STAT4, STAT2 and STAT6, and STAT3 and STAT5 (Boudinot et al., 2021), Gene loss following duplication occurrences may also restrict the gene growth. The STATs are divided into the STAT-A and STAT-B groups, with STAT1 to STAT4 being a part of STAT-A, and STAT5 and STAT6 being a part of STAT-B (Cao et al., 2021). In the present study, we observed that apart from the absence of *stat1b*, seven STAT genes have been identified in lumpfish. The host’s defense systems are greatly influenced by these genes, which are preferentially expressed in distinct cell types.

JAK proteins are crucial components of the signaling pathways of various cytokines (Bousoik and Montazeri Aliabadi, 2018), which regulate immune responses, inflammation, and intercellular communication (Morris et al., 2018). The DEG analyses from the present study (**Figure 3B**), made a basis for our model in lumpfish, where cytokines like IL*-*4/13, IL*-*2, IL*-*21, IL*-*6, IL*-*10, and IFN activate specific Janus Kinase (JAK) family receptors to transmit signals, leading to the activation of distinct STAT proteins **(** **Figure 4**). Among the JAK family members, TYK2 holds significance in mediating IL-10 dependent signaling as anti-inflammatory responses during bacterial infections. However, the mechanism by which IL-10 triggers anti-inflammatory gene expression differs markedly from the ability of IL-6 to induce inflammatory gene activity. While JAK3 is activated in response to cytokine stimulation, including IL-2 family members such as IL-4 and IL-21, it is primarily expressed in hematopoietic and immunologic cells. On the other hand, JAK2, which is more widely distributed, serves as the predominant kinase activated by approximately two-thirds of ligands (Seif et al., 2017). The significance of STAT3 in regulating immune reactions and maintaining immune homeostasis is underscored by its crucial role in both pro-inflammatory and anti-inflammatory responses (Hillmer et al., 2016). In fish, IL-6 is essential for pro-inflammatory immune responses against bacterial infections, enhancing antibody production and activating macrophages (Wang JX. et al., 2022). The IL-6/STAT3 signaling pathway potentially contributes to the expression of antimicrobial genes in infected cells (Ching et al., 2018; Wang JX. et al., 2022). This vital role of STAT3 extends to lumpfish, reaffirming its importance in immune regulation across species.

Interferons (IFN*s*) are essential for coordinating vigorous and efficient antiviral responses in vertebrates (Bonjardim, 2005), including fish (Zhang and Gui, 2012). STAT1 is especially important for innate immunity and downstream interferon (IFN) function (Skjesol et al., 2010). Both STAT1 in humans and its fish counterpart, *stat1a*, exhibit similar functions and play crucial roles in antiviral defense mechanisms. The critical role of STAT1 is believed to be conserved in fish, as demonstrated by the successful restoration of IFN-induced signaling in zebrafish *stat1* (Song et al., 2011). Upon activation, both STAT1 and *sta*t*1a* form complexes with transcription factors like IRF9, resulting in the activation of interferon-stimulated gene (ISG) expression (Lu et al., 2020; Tolomeo et al., 2022). This well-studied pathway has been extensively explored in the context of dangerous viruses such as Ebola and Severe acute respiratory syndrome coronavirus 2 (SARS-CoV-2) in humans as host (Tolomeo et al., 2022; Escudero-Perez et al., 2023). These results provide important information on the conservation and variety of the JAK-STAT pathway in fish and set the stage for future studies on the functional involvement of STAT-related genes in controlling important biological processes. It is critical to comprehend the JAK-STAT pathway’s regulatory mechanisms in fish since this reveals the pathway’s capacity to control a variety of biological activities.

Poly(I:C) is a synthetic compound that is a dsRNA mimicking viral infection and is often used to generate a type I IFN response in fish (Zhang and Gui, 2012), and other animals. In a study utilizing minipig kidney cell line, it was discovered that *stat1a* and *stat1b* were both highly upregulated after poly(I:C) stimulation, demonstrating that they had different reactions to stimulation (Wu et al., 2019). Exposure of lumpfish leukocytes to poly(I:C) led to the upregulation of *stat1a* and *stat2*. However, more research is needed to ascertain whether the underlying mechanisms for immune regulation in fish is like that in mammals, and to investigate the functional specialization of each STAT family member in fish. In chinook salmon cell line EC, *stat1b1* and *stat2* were induced with a FC > 1.5 following stimulation by salmonid recombinant type I IFN, while *stat1a1* was also induced to some extent. However, *stat1b2* was not up regulated (Dehler et al., 2019). The expression of *stat*1a paralogs was higher than *stat1b* under steady state conditions, consistent with the functional constitutive expression of *stat1a* genes observed in zebrafish (Langevin et al., 2013a; Levraud et al., 2019).

It has been observed that *stat1a* and *stat1b* expressions are linked to different tissues in fish (Xu et al., 2022). E.g., after *A. hydrophila* infection in the spleen, different expression patterns of the STAT genes were observed. At the early stages of infection, three genes, including *stat1b*, *stat5a*, and *stat6*, were markedly regulated, and two genes, *stat1a* and *stat2*, were significantly induced. Notably, the relative expression of *stat1b* in the liver, spleen, and kidney was at least 10-fold higher at the early stage of infection (Xu et al., 2022). A study found that both zebrafish and salmonid showed robust up-regulation of genes *stat1b* and *stat2*, but not of *stat1a*, in response to type I IFN induction (Langevin et al., 2013a; Levraud et al., 2019). Similarly, in a similar study involving channel catfish infected with *Edwardsiella*, *stat1b* was significantly up-regulated in the liver and intestine at 72 hpi, but it was significantly down-regulated in the intestine at 24 hpi (Jin et al., 2018). Further research is needed to confirm the consistency of fish’s regulation patterns with those of mammals and to explore the functional differentiation of each STAT family member in fish.

Different STATs have been identified in various fish species, and they play essential roles in regulating immune responses. For example, STAT1 is involved in the induction of type I IFNs and the activation of macrophages and natural killer cells. STAT3 is involved in the regulation of T cell differentiation and the production of inflammatory cytokines, while STAT5 is involved in the development of lymphoid cells and the regulation of antibody production. Additionally, STAT6 is involved in the differentiation of Th2 cells and the production of anti-inflammatory cytokines. Overall, the expression and function of different STATs in fish suggest that they play critical roles in modulating both innate and adaptive immune responses.

The identification of specific STAT-related genes in lumpfish, along with the comparison of transcriptional targets across the genome, can aid in comprehending the pathway’s regulatory mechanisms. Further studies on the functional roles of these genes in regulating vital biological processes are necessary to gain a comprehensive understanding of the JAK-STAT pathway’s significance in fish. In conclusion, this study emphasizes the importance of the JAK-STAT pathway in fish and highlights the need to gain a comprehensive understanding of its regulatory mechanisms. The study’s findings provide significant information on the conservation and diversity of the JAK-STAT pathway in fish, specifically in the lumpfish system. This research can aid in developing a foundation for further research on the functional roles of specific STAT-related genes in regulating critical biological processes in fish.

## Data availability statement

The original contributions presented in the study are included in the article/**Supplementary Material**. Further inquiries can be directed to the corresponding author.

## Ethics statement

The requirement of ethical approval was waived by Mattilsynet/The Norwegain food authority for the studies involving animals because Sampling from healthy animals do not require approval from an ethical committee in Norway. The studies were conducted in accordance with the local legislation and institutional requirements.

## Author contributions

SR: Conceptualization, performed analyses, original draft writing, and editing. PN: performed analyses HL: Conceptualization, performed analyses. GH: Mentorship, study design, funding acquisition, performed experiment, conceptualization, and drafting the manuscript for important intellectual content. All authors contributed to the article and approved the submitted version.
