# Supplementary material for: Evolutionary, comparative, and functional analyses of STATs and regulation of the JAK-STAT pathway in lumpfish upon bacterial and poly(I:C) exposure
Source: Front Cell Infect Microbiol. 2023 Sep 22;13:1252744. doi: 10.3389/fcimb.2023.1252744 (PMC10556531; doi:10.3389/fcimb.2023.1252744)
Supplement: Supplementary file 1 [file Table_1.docx]

**Supplementary Table 1. Verified STAT genes belonging to the JAK/STAT pathway in all fish species mentioned in the study.**

| **Gene** | **Human** | **Salmon** | **Zebra fish** | **Stickleback** | **Lumpfish** | **Nile tilapia*** | **Japanese medaka*** |
| --- | --- | --- | --- | --- | --- | --- | --- |
| **STAT1a** | ENSG00000115415 | ENSSSAG00000073567 | ENSDARG00000006266 | ENSGACG00000015589 | ENSCLMG00005003533 | ENSONIG00000012194  (Chr-LG23) | ENSORLG00000000162 (Chr-2) |
|  |  | ENSSSAG00000102446  ENSSSAG00000056374 |  |  |  |  |  |
| **STAT1b** |  | ENSSSAG00000093277 | ENSDARG00000076182 | ENSGACG00000002673 | - | ENSONIG00000040122  (Chr-LG16) | ENSORLG00000024182  (Chr-21) |
|  |  | ENSSSAG00000119912 |  |  |  |  |  |
| **STAT2** | ENSG00000170581 | ENSSSAG00000067332 | ENSDARG00000031647 | ENSGACG00000000707 | ENSCLMG00005010239 | ENSONIG00000019094 | ENSORLG00000028847 |
| **STAT3** | ENSG00000168610 | ENSSSAG00000003657 | ENSDARG00000022712 | ENSGACG00000008607 | ENSCLMG00005015156 | ENSONIG00000001148  (Chr-LG4) | ENSORLG00000004061  (Chr-8) |
|  |  | ENSSSAG00000010060 |  |  |  |  |  |
| **STAT4** | ENSG00000138378 | ENSSSAG00000052979 | ENSDARG00000028731 | ENSGACG00000002684 | ENSCLMG00005009987 | ENSONIG00000016817  (Chr-LG16) | ENSORLG00000015180  (Chr-21) |
|  |  | ENSSSAG00000062497 |  |  |  |  |  |
| **STAT5A** | ENSG00000126561 | ENSSSAG00000003584 | ENSDARG00000019392 | ENSGACG00000008634 | ENSCLMG00005015196 | ENSONIG00000001131  (Chr-LG4) | ENSORLG00000003961  (Chr-8) |
|  |  | ENSSSAG00000010616 |  |  |  |  |  |
| **STAT5B** | ENSG00000173757 | ENSSSAG00000048873 | ENSDARG00000055588 | ENSGACG00000015405 | ENSCLMG00005017037 | ENSONIG00000007261  (Chr-LG8) | ENSORLG00000014335 (Chr-91) |
| **STAT6** | ENSG00000166888 | ENSSSAG00000080589 | ENSDARG00000015902 | ENSGACG00000008477 | ENSCLMG00005021957 | ENSONIG00000011930 (Chr-LG20) | ENSORLG00000024001 (Chr-7) |

*Figure 5
