# Supplementary figures and images for: Evolutionary, comparative, and functional analyses of STATs and regulation of the JAK-STAT pathway in lumpfish upon bacterial and poly(I:C) exposure
Source: Front Cell Infect Microbiol. 2023 Sep 22;13:1252744. doi: 10.3389/fcimb.2023.1252744 (PMC10556531; doi:10.3389/fcimb.2023.1252744)

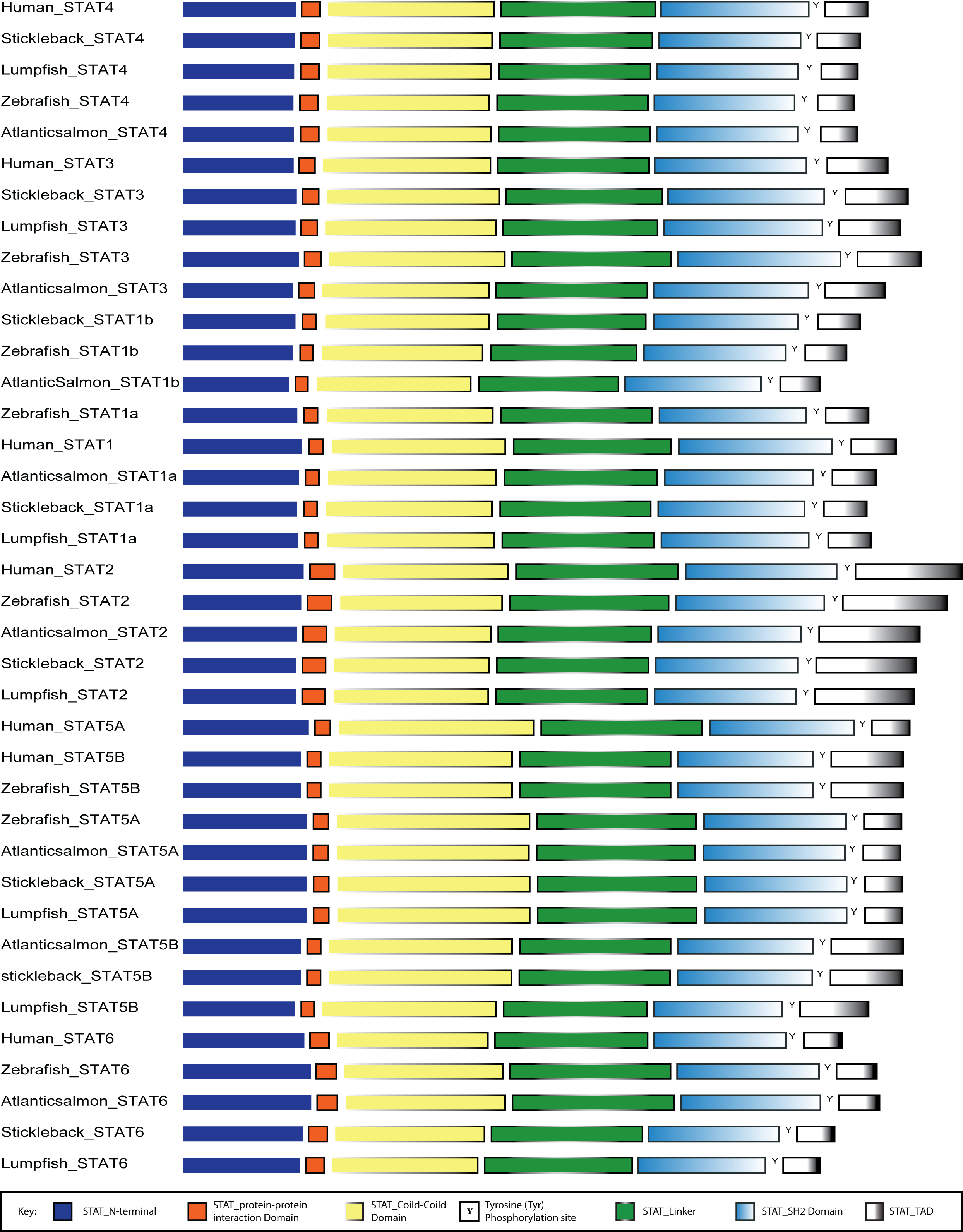

Supplement: Supplementary file 2 [file Image_1.tif]
